# Updated Smoke Exposure Estimate for Indonesian Peatland Fires Using a Network of Low‐Cost PM_2.5_ Sensors and a Regional Air Quality Model

**DOI:** 10.1029/2024GH001125

**Published:** 2024-11-03

**Authors:** Ailish M. Graham, Dominick V. Spracklen, James B. McQuaid, Thomas E. L. Smith, Hanun Nurrahmawati, Devina Ayona, Hasyim Mulawarman, Chaidir Adam, Effie Papargyropoulou, Richard Rigby, Rory Padfield, Shofwan Choiruzzad

**Affiliations:** ^1^ School of Earth and Environment University of Leeds Leeds UK; ^2^ National Centre for Earth Observation University of Leeds Leeds UK; ^3^ Department of Geography and Environment London School of Economics and Political Science London UK; ^4^ Department of International Relations Universitas Indonesia Kota Depok Indonesia; ^5^ University of Palangka Raya Palangka Raya Indonesia

**Keywords:** low‐cost sensor, PM_2.5_, air pollution, peat fires, El Niño, health

## Abstract

Indonesia accounts for more than one third of the world's tropical peatlands. Much of the peatland in Indonesia has been deforested and drained, meaning it is more susceptible to fires, especially during drought and El Niño events. Fires are most common in Riau (Sumatra) and Central Kalimantan (Borneo) and lead to poor regional air quality. Measurements of air pollutant concentrations are sparse in both regions contributing to large uncertainties in both fire emissions and air quality degradation. We deployed a network of 13 low‐cost PM_2.5_ sensors across urban and rural locations in Central Kalimantan and measured indoor and outdoor PM_2.5_ concentrations during the onset of an El Niño dry season in 2023. During the dry season (September 1st to October 31st), mean outdoor PM_2.5_ concentrations were 136 μg m^−3^, with fires contributing 90 μg m^−3^ to concentrations. Median indoor/outdoor (I/O) ratios were 1.01 in rural areas, considerably higher than those reported during wildfires in other regions of the world (e.g., USA), indicating housing stock in the region provides little protection from outdoor PM_2.5._ We combined WRF‐Chem simulated PM_2.5_ concentrations with the median fire‐derived I/O ratio and questionnaire results pertaining to participants' time spent I/O to estimate 1.62 million people in Central Kalimantan were exposed to unhealthy, very unhealthy and dangerous air quality (>55.4 μg m^−3^) during the dry season. Our work provides new information on the exposure of people in Central Kalimantan to smoke from fires and highlights the need for action to help reduce peatland fires.

## Key Points

- Mean outdoor PM_2.5_ concentrations during the dry season were 136 μg m^−3^ and fires contributed ∼90 μg m^−3^ to PM_2.5_ concentrations
- I/O ratios indicate that housing stock provides little protection from outdoor PM_2.5_
- 1.62 million people in Central Kalimantan were exposed to unhealthy, very unhealthy and dangerous air quality (>55.4 μg m^−3^) during the dry season

## 1 Introduction

Indonesian peatlands account for more than 35% of the world's tropical peat, and between 8% and 15% of total land cover in Indonesia (Xu et al., 2018). In pristine tropical peatlands water levels remain above the surface for much of the year (Taufik et al., 2018), meaning they are resilient to fires (Evers et al., 2017). Large areas of Indonesian peatlands have been altered by deforestation and drainage (via canals), for logging and conversion to plantation (Dohong et al., 2017; Miettinen et al., 2016) lowering water levels and increasing their susceptibility to fire, especially during El Niño years and in periods of drought (Konecny et al., 2016; Putra et al., 2018; Taufik et al., 2018).

Indonesian peat fires have important impacts on forest ecosystems (Harrison et al., 2024) and release large quantities of carbon dioxide and air pollutants to the atmosphere resulting in substantial economic damages (Kiely et al., 2021). Vegetation fires on peatland can burn down into the peat below the surface (Roulston et al., 2018) and emissions from peat burning dominate total fire emissions (Heil et al., 2007). Estimates of fire emissions have large uncertainties associated with them (Hu et al., 2018; Liu et al., 2020) and uncertainties are particularly large for peat fires. Uncertainties stem from difficulty in detecting tropical peat fires due to frequent cloud cover and low burning temperatures (Ge et al., 2014), and therefore underestimating burned area. In addition, emissions from peat fires are determined by the depth which fires burn, which is highly variable and poorly constrained (Huang & Rein, 2015; Simpson et al., 2016). Emission factors (EF) of peat fires are much higher than vegetation fires due to the dominance of inefficient smouldering combustion (Smith et al., 2018), however there are few measurements of peat fire emission factors (EFs) in Indonesia and the EFs which do exist are highly variable (Kiely et al., 2019; Santoso et al., 2022).

Emissions from Indonesian fires expose large populations in the region to poor air quality (Crippa et al., 2016; Kiely et al., 2019, 2020). In 2015, Indonesian fires exposed an estimated 20 million people to daily PM_2.5_ concentrations exceeding 150 μg m^−3^ (Kiely et al., 2020). A lack of ground‐based air pollution monitoring close to the fires limits the opportunity to evaluate modeled air pollutant concentrations (e.g., Crippa et al., 2016; Kiely et al., 2019, 2020). New measurements of air pollution close to Indonesian fires are needed to improve understanding of the air pollution degradation caused by peat fires.

Smoke contains many chemicals that are harmful to human health (Naeher et al., 2007). In particular, PM_2.5_, which is associated with increases in mortality and morbidity (Pope & Dockery, 2006). Health impact assessments (HIA) are widely used to quantify the impacts of exposure to PM_2.5_. Health impact assessments rely on concentration response functions that are derived from cohort studies, which are heavily biased toward the west (Burnett et al., 2018; Chen & Hoek, 2020; Pope III et al., 2020). The cohort studies follow populations over long time periods (decades) and relate outdoor air pollution concentrations to health impacts observed. Since the population moves between indoor and outdoor environments, the indoor to outdoor (I/O) ratio and fraction of time spent indoors and outdoors is important. I/O ratios are determined by how well sealed a building is from outside air and how many indoor sources of air pollution there are. An I/O ratio <1 indicates air pollution concentrations indoors are lower than outdoors, and therefore buildings are well sealed from outside air pollution and there are few indoor sources of air pollution. An I/O ratio >1 indicates air pollution concentrations indoors are higher than outdoors, meaning there are important indoor sources of air pollution. Studies that have measured I/O ratios in locations affected by fires have been largely focussed on the USA. In the USA, household I/O ratios range between 0.23 and 0.88 (Barn et al., 2008; He et al., 2022; Henderson et al., 2005; Kirk et al., 2018; May et al., 2021). While in other settings, like commerce and schools, I/O ratios are generally higher at between 0.58 and 0.91 (May et al., 2021; Stampfer et al., 2024). In contrast, a study focussed on Palangkaraya, Indonesia found the mean I/O ratio during the dry season in 2019, an El Niño high‐fire year, ranged between 0.83 and 1.25 (Ardiyani et al., 2023). This highlights that the I/O ratio in USA may not be representative of other regions of the world where buildings are more poorly sealed. In addition, given the range of I/O ratios seen across different environments (e.g., commerce, schools, households), it is also important to consider how much time is spent in different environments. Several studies have investigated activity budgets (time spent doing individual activities in a 24‐hr period) and how this could affect exposure. For example, the National Human Activity Pattern Survey in the USA (Klepeis et al., 2001) indicated that 87% of respondents' time was spent in enclosed buildings, with a further 6% of their time spent in enclosed vehicles. The results highlight the large fraction of time people spend indoors and the importance of knowing indoor PM_2.5_ concentrations to better estimate population exposure.

This work aims to address some of the current limitations in air quality impact assessments for Indonesian peat fires. In August 2023, during the onset of an El Niño dry season, we deployed a network of 13 low‐cost PM_2.5_ sensors across 7 urban/rural locations and 1 remote location in Central Kalimantan. PM_2.5_ sensors were deployed inside and outside of households, to provide information on indoor and outdoor concentrations of PM_2.5_. To estimate exposure, this information was combined with results from a questionnaire on time spent in different micro‐environments and whether these were indoor or outdoor spaces (Table S1 in Supporting Information S1). To estimate exposure across Central Kalimantan we combined modeled ambient outdoor PM_2.5_ concentrations with our measured I/O ratios and information on our participants' time spent indoors and outdoors.

## 2 Method

**Figure 1:**
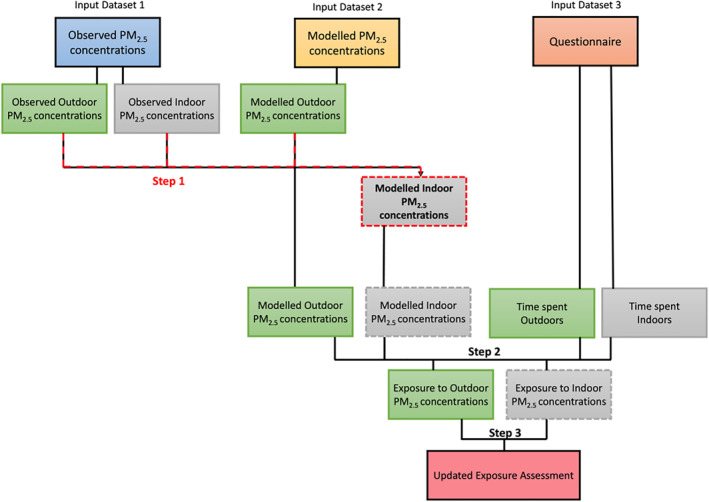
Data sets and method used in this study to generate an updated exposure assessment. The input data sets used are: (Input Data set 1) Observed ambient indoor and outdoor PM_2.5_ concentrations from Purple Air monitors (Indoor and Outdoor), (Input Data set 2) Modeled ambient outdoor PM_2.5_ concentrations from WRF‐Chem (Outdoor only), (Input Data set 3) time spent indoors and outdoors (Indoor and Outdoor), collected from our participants' questionnaires. An updated exposure assessment is calculated using 3 steps: (Step 1) The ratio between observed indoor and outdoor PM_2.5_ concentrations (input Data set 1) is combined with modeled outdoor PM_2.5_ concentrations (input Data set 2) to calculate modeled regional indoor PM_2.5_ concentrations. (Step 2) Modeled indoor and outdoor PM_2.5_ concentrations are combined with time spent indoors and outdoors (input Data set 3) to calculate exposure to PM_2.5_ indoors and outdoors. (Step 3) This is combined to calculate the updated exposure assessment, which represents overall exposure.

The data sets and method used in the paper are summarized in Figure 1.

### 2.1 Measurements of PM_2.5_ Using Low‐Cost PM_2.5_ Sensors

**Figure 2:**
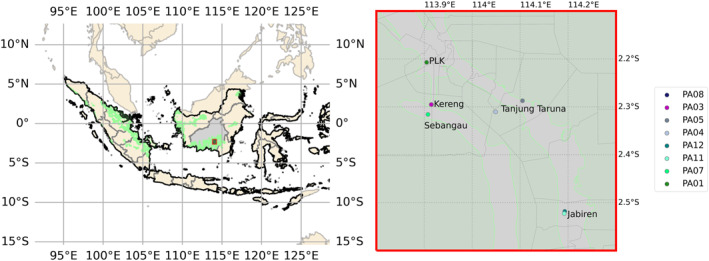
WRF‐Chem modeling domain covering Indonesia with Central Kalimantan in gray and peatland shaded in green. The field study domain in Central Kalimantan within the modeling domain is also indicated in red. Locations of outdoor Purple Air sensors in the field study domain are shown on the right (see Table 1 for more information), with location names for reference (PLK = Palangkaraya).

We deployed a network of 13 low‐cost Purple Air PA‐II sensors across 8 locations in Pulang Pisau, Central Kalimantan (Table 1, Figure 2), which measured PM_2.5_ concentrations between August 16th and 1st December 2023. Pulang Pisau was chosen as the region is home to deep, degraded peatlands and is prone to high fire activity during drought conditions (e.g., El Niño years) (Figure S4 in Supporting Information S1). The sensors were deployed at a combination of households (5), village offices (1), a hospital (1) and a remote forest location (1). At each location a sensor was mounted indoors, in the living room ∼1–2 m from the floor, and another sensor was mounted outdoors, in a sheltered location ∼1–2 m from the ground (except JBR_03, JBR_04 and SBG_01 where sensors were only deployed outdoors). The locations for the sensors were decided based upon the volunteers that were recruited by the village head in each location. All houses are wooden (except PLK_01, which is concrete), while the village office and hospital are concrete. All buildings have open vents above the doors and windows, meaning they are poorly sealed from outdoor air. Five locations reported having a resident smoker and frequency of smoking ranged from 5 to 32 cigarettes per day (Table 1).

**Table 1:** Inventory of Indoor and Outdoor Purple Air Sensors Deployed in Central Kalimantan, Indonesia Between August 16th and December 1st

Village	Location ID	Indoor sensor ID	Outdoor sensor ID	Building type	Location type	Smoking
Kereng	KR_05	PA06	PA08	Village Office	Urban	Yes
Kereng	KR_06	PA10	PA03	Village Hospital	Urban	No
Tanjung Taruna	TT_03	PA02	PA05	Household	Rural	Yes
Tanjung Taruna	TT_05	PA13	PA04	Household	Rural	Yes
Jabiren	JBR_03		PA12	Household	Rural	Yes
Jabiren	JBR_04		PA11	Household	Rural	Yes
Palangkaraya	PLK_01	PA09	PA01	Household	Urban	No
Sebangau	SBG_01		PA07	Forest	Remote	No

*Note.* Locations where no indoor Purple Air sensor was deployed are blank. The sensor identification number (Indoor Sensor ID/Outdoor Sensor ID), building type, type of location and whether there is a smoker present at the address are also indicated.

The PA‐II sensors report PM_2.5_ concentrations at 2‐min time resolution (averaged from 1 s samples). The PA‐II sensors use two Plantower PMS5003 laser particle counters to calculate the size of particles. The sensors draw air in and past the light path at a flow rate of 0.1 L min^−1^. Particle size is calculated using Mie theory and a photodiode detector that converts scattered light into a voltage pulse. Particle counts are split into 6 size bins (0.3, 0.5, 1, 2.5, 5 and 10 μm) and an algorithm provided by Purple Air is used to convert particle counts into mass concentrations for PM_2.5_ and PM_10_ (both in μg m^−3^). The mass concentration range of the sensors is 0–500 μg m^−3^ with a mass concentration accuracy of ±10 μg m^−3^ between 0 and 100 μg m^−3^ and ±10% between 100 and 500 μg m^−3^. Mass concentrations are provided for two different particle‐count to mass concentration conversions: (a) CF_1 which uses the “average particle density” for indoor particulate matter and (b) CF_ATM which uses the “average particle density” for outdoor particulate matter. In both cases the particle density lies between 1 and 2 kg m^−3^. For both the indoor and outdoor sensors we use CF_1 since the relative humidity adjustment we apply was developed for this metric (Section 2.3.1). The sensors work effectively in a temperature range of −10 to 60°C and 0%–99% relative humidity. Accuracy assessments of the Plantower PMS5003 particle counters have found them to perform well against regulation certified air quality monitoring equipment, once corrected for variability in relative humidity (Chan et al., 2021).

#### 2.1.1 Relative Humidity Adjustment

Previous work has shown that low‐cost sensors can begin to overestimate observed PM_2.5_ concentrations at relative humidity (RH) above 50% due to hygroscopic growth of particles (Jayaratne et al., 2018; Magi et al., 2019; Nilson et al., 2022; Zamora et al., 2019). We follow the Quality Control procedures and a relative humidity adjustment developed by Nilson et al. (2022). Nilson et al. (2022) compared multiple RH corrections and we use their “RH Growth” correction model (Equation 1), which has the best performance at moderate to high PM_2.5_ concentrations, which are important for health, and which we are likely to see during fires.

(1) $$\text{Adjusted}\;{\text{PM}}_{2.5}={\text{PM}}_{2.5\;(\text{CF}=1)}\;/\;1+\left(\frac{0.24}{\frac{100}{\text{RH}}-1}\right)$$

In Equation 1 “adjusted PM_2.5_” is the PM_2.5 (CF=1)_ concentration that has been adjusted for relative humidity. PM_2.5 (CF=1)_ is the PM_2.5_ concentration calculated using the “average particle density” for indoor particulate matter. RH is the relative humidity as measured by the Purple Air Monitor (in %).

Following Nilson et al. (2022), the RH measured by the Purple Air monitors was restricted to 30%–70% and any values above or below of this range were set to 30% and 70% respectively. By restricting RH to 30%–70%, the overcorrection of PM_2.5_ measurements at extreme RHs is avoided. Across sites, no monitors had missing RH observations (where there were PM_2.5_ measurements). However, 78% of observations had RH higher than 70% (replaced with a value of 70%) and no observations had a RH less than 30%. The mean reduction in observed PM_2.5_ across all sites when the RH adjustment was applied was 20 μg m^−3^ (min: 2 μg m^−3^, max: 66 μg m^−3^) for indoor sensors and 91 μg m^−3^ (min: 5 μg m^−3^, max: 227 μg m^−3^) for outdoor sensors.

#### 2.1.2 Quality Control

Following Nilson et al. (2022), we compared the PM_2.5_ concentrations for Channel A and Channel B to identify any failures from individual sensors (channels) within each monitor. We flagged hours where the error in PM_2.5_ concentrations was >50% of the mean PM_2.5_ concentrations from both sensors. In most cases, except PA03 at KR06, both Plantower PMS5003 laser particle counters (Channel A and B) inside Purple Air monitors gave very similar PM_2.5_ readings throughout the study period. However, Channel A within PA03 (the outdoor sensor at KR06) substantially deviated from Channel B on September 26th at 22:00 UTC, reaching concentrations >3,000 μg m^−3^. PM_2.5_ concentrations remained >1,000 μg m^−3^ until the end of the study period. This issue has been previously reported in the literature and is believed to be due foreign objects (e.g., insects, dust) within the sensor. Despite this, PA03 Channel B PM_2.5_ concentrations remained in close agreement with PM_2.5_ concentrations from the indoor sensor at the same location (PA10), as well as indoor and outdoor sensors a nearby site (PA08 and PA06 at KR05) (Table 1). Thus, indicating that data from Channel B is reliable despite the issues with Channel A. Therefore, we chose not to exclude PA03 from the study. For all sites sensors except PA03 we present the mean of Channel A and Channel B, while for PA03 we only present Channel B.

Hourly mean PM_2.5_ concentrations were calculated if >75% of 2‐min data within a given hour was available, otherwise the hour was flagged. We followed the same protocol to create daily means, flagging days where <75% of hourly data was available.

#### 2.1.3 Government Monitoring Sites

Daily‐mean measurements of PM_2.5_ between August 16th and 1 December 2023 were taken for 7 government monitoring sites in Indonesian Borneo (https://www.bmkg.go.id/kualitas‐udara/informasi‐partikulat‐pm25.bmkg). Currently, the Indonesian Ministry of Environment and Forestry (KLHK) utilize the Air Quality Monitoring System (AQMS) equipped with Horiba Air Pollution Dust Analyzer (APDA)‐371 as the FRM (HORIBA) for PM_2.5_ monitoring (Kurniawati et al., 2024). Beta‐ray attenuation is used to measure PM_2.5_ concentrations. Hourly measurements are taken. Data is provided at daily mean time resolution, but no documentation is available on data quality procedures.

#### 2.1.4 Purple Air Evaluation

We compared daily‐mean PM_2.5_ concentrations from the outdoor Purple Air sensor located in Palangkaraya (PA01) to a reference grade sensor in Palangkaraya, located within 2 km. Daily‐mean PM_2.5_ concentrations measured by PA01 are in good agreement (*r*: 0.92, NMBF: 0.05, NMAE: 0.28, RMSE: 51.13 mg m^−3^) with PM_2.5_ concentrations measured by the reference grade sensor during the dry season. Therefore, the PM_2.5_ concentrations measured by the network of Purple Air sensors that we deployed can be used to quantify the impacts of fires on PM_2.5_ concentrations during the dry season across Central Kalimantan.

### 2.2 Modeled PM_2.5_ Concentrations

We simulated hourly PM_2.5_ concentrations for August 1st to December 31st using the Weather Research and Forecasting model coupled to Chemistry (WRF‐Chem) model (version 4.2), a fully coupled atmospheric chemistry model, at 30 km horizontal resolution. We focused on the dry season (August 1st–October 31st), when fires occur, and simulate the wet season period (November 1st–December 31st) to represent ambient conditions for the rest of the year (1 January 2023 to 31 July 2023 and 1 November 2023 to 31 December 2023). We used the same model domain as Kiely et al. (2019, 2020) (Figure 2), which covers much of Indonesia, and south‐east Asia but excludes West Papua. Model simulations were at 30 km resolution, with 33 vertical levels (extending from the surface up to 10 hPa). The contribution of fires to PM_2.5_ concentrations was calculated by comparing two model scenarios, with and without fires (PM_2.5_from_fires_ = PM_2.5_fires_–PM_2.5_no_fires_).

#### 2.2.1 Meteorology

Meteorology was initialized using European Center for Mid‐range Weather Forecasting Reanalysis v5 (ERA5) at 6‐hourly temporal resolution, 0.1° spatial resolution, over 38 pressure levels (Hoffmann et al., 2018). Nudging of potential temperature, the horizontal and vertical winds and the water vapor mixing ratio was only performed above the boundary layer.

#### 2.2.2 Chemical Boundary Conditions

Chemical boundary conditions are provided by the Whole Atmosphere Community Climate Model (WACCM) 6‐hourly simulation data (Marsh et al., 2013; UCAR, 2020a) with spatial resolution of 0.9 × 1.25° and 88 vertical levels (UCAR, 2020b). Whole Atmosphere Community Climate Model meteorology is driven by the NASA Global Modeling and Assimilation Office Goddard Earth Observing System Model (GEOS‐5) model. Anthropogenic emissions for 2014 are from the Community Emissions Data System and fire emissions from the Fire Inventory from NCAR (FINN) version 1 (v1) are used in WACCM.

#### 2.2.3 Anthropogenic Emissions

EDGAR‐HTAP_v3 mosaic anthropogenic emissions for 2018 at 0.1° resolution are used (Crippa et al., 2023). We subsequently added sector specific diurnal cycles to the emissions, using diurnal cycles from Olivier et al. (2003).

EDGAR‐HTAP_v3 consists of a global, gridded, air pollution emission inventory compiled using a mosaic of officially reported, national gridded inventories. Anthropogenic emissions for most of Asia are from the Regional Emission Inventory in Asia (REAS) inventory version 3.2.1. Anthropogenic emissions include SO_2_, NO_x_, CO, NMVOC, NH_3_, PM_10_, PM_2.5_, BC, and OC. All anthropogenic emissions are included, except large‐scale biomass burning (e.g., wildfires). EDGAR‐HTAP_v3 provides extended temporal coverage of air pollutant emissions, as well as improved sectoral and geographical coverage compared with EDGAR‐HTAP_v2.

#### 2.2.4 Chemistry Scheme

The Model for Ozone and Related Chemical Tracers, version 4 (MOZART‐4) (Emmons et al., 2009) was used to calculate gas‐phase chemical reactions. While the Model for Simulating Aerosol Interactions and Chemistry (MOSAIC) scheme is used to represent aerosol chemistry and physics, with sub‐grid scale aqueous chemistry (Zaveri et al., 2008). Four sectional discrete size bins (0.039–0.156, 0.156–0.625, 0.625–2.5, and 2.5–10) are used to represent aerosols. The combination of the MOSAIC scheme and four size bins balances detailed chemistry with computational expense.

### 2.3 Fire Emissions

We generated daily fire emissions at 1 km resolution using the FINNpeatSM method previously developed, and described in detail, by Kiely et al. (2019). We used 2023 fire emissions from the daily FINNv1_nrt product (1 km resolution), as previously used and evaluated in Graham et al. (2021), to generate the FINNpeatSM emissions.

In brief, FINNpeatSM adds below‐ground burning of peatland to the FINN emissions, which previously only included above‐ground vegetation fires. FINNpeatSM assumes that when MODIS fire hotspots are detected on peatland (World Resources Institute, 2017) the fire burns into the peat below. Emissions are calculated using Equation 2:

(2) $$E_{s}=\text{BA}\times \text{BD}\times \rho \times {\text{EF}}_{s}$$

*E*_*s,*_ the emissions of a species, *s*, for a given fire is calculated as the product of the burned area (BA), the burn depth (BD), the fuel density (peat density in this case) (*p*) and the emission factor for species, *s*, (EF_*s*_).

#### 2.3.1 Burned Area

Like Kiely et al. (2019) the burned area of peat fires was assumed to be smaller than above‐ground surface fires. For above‐ground surface fires, a burned area of 100 ha is assumed, however, for below‐ground peat fires, burned area was assumed to be 40 ha (Tansey et al., 2008).

#### 2.3.2 Soil Moisture

Kiely et al. (2019) used daily soil moisture from the European Space Agency (ESA CCI SMv04.4), which was averaged to 2‐degree resolution to create a spatially complete map of soil moisture (Dorigo et al., 2017; Gruber et al., 2017; Liu et al., 2012). We updated this method to use NASA's Level 4 Soil Moisture Active Passive Product (SMAP) since the spatial (9 km) and temporal resolution (3‐hourly) is much higher. The Level 4 data merges SMAP measurements of soil moisture in the top 5 cm of the soil column with estimates from a land‐surface model to provide soil moisture in the top 1 m of the soil column. The land‐surface model is driven with meteorological reanalysis and includes soil moisture transfer between the surface and root zones (up to 1 m depth). The SMAP is both spatially and temporally complete, so we aggregated the 3‐hourly data to daily‐mean values. Following Kiely et al. (2019), we used the SMAP data to linearly scale burn depth between a minimum burn depth of 5 cm, when soil moisture is high, and a maximum burn depth of 37 cm, when soil moisture is low. Kiely et al. (2019) used a high soil moisture threshold of 0.25 m^3^ m^−3^ and a low soil moisture threshold of 0.15 m^3^ m^−3^, finding these thresholds gave the closest match between modeled and observed PM_2.5_ concentrations. However, the soil moisture product used by Kiely et al. (2019) was much coarser and observed PM_2.5_ was from monitoring sites much further from the fires. Due to the increased spatial resolution of SMAP there is more spatial variability in soil moisture.

In addition, observations from the Purple Air sensors provide PM_2.5_ concentrations close to fires (as shown in Figure S4 in Supporting Information S1). Therefore, we iterate over high and low soil moisture values for 2023 to find the combination that resulted in the closest match between modeled and observed PM_2.5_ concentrations. The soil moisture threshold combinations we tested, and the dry season model evaluation for each, are given in Table 3 and shown in Figure S2 in Supporting Information S1 (see Model Evaluation for evaluation).

#### 2.3.3 Fuel Density and Emissions Factors

We used the same peat density (0.11 g cm^−3^) and emission factors as Kiely et al. (2019). For PM_2.5_ the emission factor used is 22.3 g kg^−1^.

### 2.4 Updated Exposure Assessment

#### 2.4.1 Purple Air and WRF‐Chem PM_2.5_ Concentrations

The median ratio between indoor and outdoor PM_2.5_ concentrations (I/O ratio, Equation 3) from Purple Air data is combined with modeled ambient outdoor (Mod_outdoor_) PM_2.5_ concentrations from WRF‐Chem (Equation 4) to estimate modeled indoor (Mod_indoor_) PM_2.5_ concentrations.

(3) $$I/O\;ratio=\frac{{Obs\;}_{indoor}}{{Obs}_{outdoor}}$$

(4) $${\mathrm{Mod}}_{indoor\;}={\mathrm{Mod}}_{outdoor\;}\times I/O\;ratio$$

Finally, an updated exposure assessment for the Central Kalimantan is calculated by combining modeled indoor and outdoor PM_2.5_ concentrations with the average amount of time spent indoors and outdoors across all villages (collected using a questionnaire (Table S1 in Supporting Information S1)).

#### 2.4.2 Questionnaire (Time Spent Indoors and Outdoors)

Volunteers who were recruited from each location participated in a short questionnaire during the sensor deployment (Table S1 in Supporting Information S1). The questionnaire aimed to provide context on exposure of the volunteers and included questions on socioeconomic status, housing materials, sources of air pollution, such as smoking and cooking, and the average time which the volunteers spent indoors and outdoors in a 24‐hr period. Each volunteer was given 24 pebbles that they could spend between different locations: home and their three main livelihoods (jobs) (e.g., 10 pebbles (hours) at home) (Camfield & Ruta, 2007). Once the volunteers had spent their pebbles at each location, they were asked to split the pebbles into how much time they spent indoors and outdoors at each of these locations (e.g., 10 pebbles (hours) at home: 8 pebbles (hours) indoors, 2 pebbles (hours) outdoors). This data was used to quantify the time each volunteer spent indoors and outdoors (by summing across time indoors/outdoors at home and all livelihoods).

#### 2.4.3 Combining Modeled Indoor and Outdoor PM_2.5_ With Questionnaires

To account for the average exposure of the population, the average modeled indoor and outdoor PM_2.5_ concentration (as calculated in Equation 4) was weighted by the average amount of time that volunteers stated they spent indoors or outdoors (Equation 5).

(5) $$\mathrm{Exp}osure=\sum_{i=1}^{N}{conc}_{e}\times \frac{t_{e}}{24}$$

Where N (=number of micro‐environments)–in this study *N* = 2.

conc_e_ is the modeled PM_2.5_ concentration in a particular environment (e), *t*_e_ is the number of hours each day spent in a particular environment (e), and 24 is the total number of hours in each day. Therefore, exposure is the total modeled PM_2.5_ concentration a population is exposed to, via the total number of environments (N) they spend their time in.

These findings were scaled across the region of Central Kalimantan to account for indoor and outdoor population exposure. The method has several assumptions. Firstly, that the sample locations in each settlement are representative of the region. The sample size in each location in this study is small and volunteers were chosen by the village head so may not be random. The non‐random volunteer selection could introduce bias into the study as we assume activity patterns indoors and outdoors are representative of the broader population in Central Kalimantan, which may not be true. Since we cannot say whether the data collected is representative of other islands in Indonesia, such as Java or Sumatra, we do not apply the method to other areas of Indonesia. Secondly, since the sample is small, we cannot be sure we have covered all potential livelihoods in each village and so may under or overestimate the time spent inside/outside. Thirdly, our air quality model simulates PM_2.5_ concentrations over a 30 km grid and there are issues when comparing against measurements made at a specific location. This model resolution is likely to be sufficient for simulating regional air quality issues, such as that arising from peatland fires. Previous studies of the impacts of Indonesian fires have applied similar scales and also perform model evaluation using ground‐based monitoring sites (e.g., Crippa et al., 2016; Kiely et al., 2019, 2020; Roberts & Wooster, 2021). Therefore, despite these limitations, the method is likely to give a more realistic representation of the population's exposure to PM_2.5_ from peat fires in Central Kalimantan than using outdoor ambient modeled concentrations alone.

##### 2.4.3.1 Time‐Periods Used for Comparisons

We refer to several time‐periods in the results, these are the pre‐dry season (August 16th–August 31st), the dry season (September 1st to October 31st) and the wet season (November 1st to December 1st). We also refer to the fire‐derived PM_2.5_ during comparisons. This is the dry season PM_2.5_ concentration minus the mean PM_2.5_ concentration in the wet season.

## 3 Results and Discussion

### 3.1 Outdoor and Indoor Daily Mean PM_2.5_ Concentrations

We use our network of Purple Air PM_2.5_ sensors deployed across the Pulang Pisau region of Central Kalimantan to characterize the impact of peat fire smoke on PM_2.5_ concentrations across the region, close to the source of fires. In addition, since sensors were deployed both indoors and outdoors, we can quantify I/O ratios to provide improved information on exposure of the population.

#### 3.1.1 PM_2.5_ Concentrations

**Figure 3:**
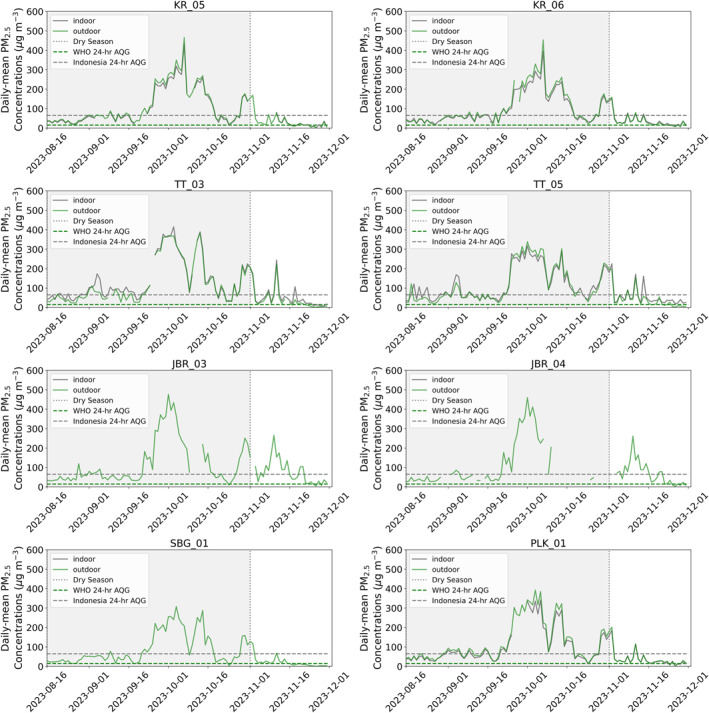
Daily mean PM_2.5_ concentrations (μg m^−3^) between 16th August 2023 and 1st December 2023 from each Purple Air monitoring location (details in Table 1). Daily‐mean indoor (green) and outdoor (gray) PM_2.5_ concentrations are shown. The World Health Organization 24‐hr guideline limit (15 μg m^−3^) (dashed green line) and the Indonesian 24‐hr guideline limit (dashed gray line) are shown. The dry season is indicated in gray shading.

In the pre‐dry season, indoor and outdoor PM_2.5_ concentrations were similar at all sites, with average PM_2.5_ concentrations of 46 μg m^−3^ indoors and 39 μg m^−3^ outdoors. PM_2.5_ concentrations at all sites were well above the WHO 24‐hr air quality guideline (15 μg m^−3^), but only exceeded the Indonesian 24‐ hour air quality guideline (65 μg m^−3^) on 1–3 days (Figure 3). In general, during this period, variability in daily mean PM_2.5_ concentrations was low across all sites. However, some isolated, local peaks in PM_2.5_ concentrations were evident at individual sites, when concentrations reached >120 μg m^−3^ (e.g., TT_05). This is likely due to local pollution sources such as trash burning, which is commonplace in the region (Irianti & Prasetyoputra, 2018). Across urban (KR_05, KR_06 and PLK_01) locations, indoor PM_2.5_ concentrations (37 μg m^−3^) were lower than outdoor PM_2.5_ concentrations (40 μg m^−3^). In contrast, in rural (TT_03 and TT_05) locations, indoor PM_2.5_ concentrations (59 μg m^−3^) were higher than outdoor (43 μg m^−3^) concentrations. In rural areas, both indoor and outdoor PM_2.5_ concentrations were higher than in urban locations.

During the dry season indoor and outdoor concentrations increased homogenously across all sites due to smoke from peatland fires (Figure 3). During this period mean indoor PM_2.5_ concentrations were 133 μg m^−3^ but varied between 13 and 433 μg m^−3^. Mean outdoor PM_2.5_ concentrations were higher (136 μg m^−3^ and range from 4 to 502 μg m^−3^). Both indoor and outdoor PM_2.5_ concentrations in urban locations (123 μg m^−3^ and 136 μg m^−3^, respectively) were generally lower than in rural areas (147 μg m^−3^ and 145 μg m^−3^, respectively). The results demonstrate the regional impact of peat fires on air quality. PM_2.5_ emissions from peat fires are emitted close to the surface for weeks to months. Emissions are then transported around the region under slack, easterly flow, increasing PM_2.5_ concentrations across the region homogenously. The results also suggest that across all locations it is very difficult for the population to reduce their exposure to PM_2.5_, since indoor concentrations were also well above the WHO and Indonesian guideline limits.

During the wet season, mean indoor and outdoor PM_2.5_ concentrations returned to pre‐dry season levels at all sites (43 μg m^−3^ and 46 μg m^−3^, respectively) (Figure 3). As in the pre‐dry season, urban indoor and outdoor PM_2.5_ concentrations (34 μg m^−3^ and 37 μg m^−3^, respectively) were considerably lower than in rural locations (56 μg m^−3^ and 34 μg m^−3^, respectively).

Overall, these results indicate that there may be differences in indoor air pollution between different socioeconomic groups. But more observations of hourly/daily PM_2.5_ concentrations are required to fully understand the impact of air pollution on different socioeconomic groups and related indicators, for example, respiratory illness, mental health impacts, days of education lost from fire events, and so forth.

Finally, we used PM_2.5_ concentrations in Sebangau Forest (SBG_01) (Figure 3), a remote site with lower influence from other anthropogenic emissions but with a similar influence from fire emissions, to isolate the impacts of fire PM_2.5_ on PM_2.5_ concentrations. Mean PM_2.5_ concentrations at this site were 29 μg m^−3^ during the pre‐dry season, ∼10–15 μg m^−3^ lower than any of the other sites. PM_2.5_ concentrations increased in September at Sebangau, peaking at >300 μg m^−3^ in the dry season. The peak in PM_2.5_ concentrations occurred at the same times across all sites, indicating the increase is likely due to fire‐derived PM_2.5_. Average dry season PM_2.5_ concentrations were 106 μg m^−3^ at Sebangau. PM_2.5_ concentrations return to pre‐dry season values (23 μg m^−3^) through November, indicating the end of the fires. Thus, supporting our previous findings that fires contributed ∼85–90 μg m^−3^ to PM_2.5_ concentrations across the region.

**Figure 4:**
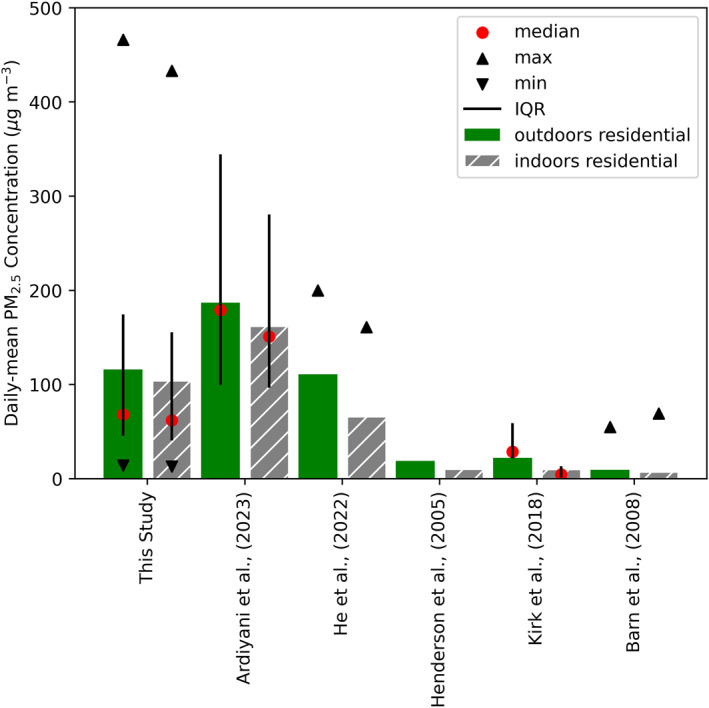
Comparison of observed daily‐mean indoor and outdoor PM_2.5_ concentrations between this study (Kereng and Palangkaraya only) and previous wildfire smoke studies in residential settings. Bars indicate the mean PM_2.5_ concentration, outdoor concentrations are in green and indoor concentrations are in gray with hatching. The median is indicated by red circles, the interquartile range by the black bar, and maximum and minimum PM_2.5_ concentration are indicated by black triangles.

We compare the observed indoor and outdoor daily‐mean PM_2.5_ concentrations from Kereng and Palangkaraya with previous studies (Figure 4). Our observed indoor and outdoor PM_2.5_ concentrations are similar to Ardiyani et al. (2023), who also deployed sensors in Palangkaraya, Central Kalimantan, Indonesia during El Niño drought conditions in 2019. The 25th–75th percentiles (IQR) overlap, however, mean observed indoor and outdoor daily‐mean PM_2.5_ concentrations are ∼100 μg m^−3^ higher in Ardiyani et al. (2023), likely because fires in 2019 were more severe. All other studies shown were based in the USA and, in all studies, except He et al. (2022), mean outdoor and indoor PM_2.5_ concentrations are considerably lower than our study or Ardiyani et al. (2023). Mean outdoor PM_2.5_ concentrations in He et al. (2022), are similar to our study (110 μg m^−3^ compared with 116 μg m^−3^ in this study), however maximum observed PM_2.5_ concentrations in our study are >250 μg m^−3^ higher, indicating peak outdoor PM_2.5_ concentrations are considerably higher. In our study mean indoor PM_2.5_ concentrations are 10% lower than mean outdoor concentrations, similar to the 13% reduction in Ardiyani et al. (2023). In the USA, mean indoor concentrations are 23%–55% lower than outdoor concentrations. This suggests that buildings may provide better protection from outdoor air pollution in the USA than Indonesia.

#### 3.1.2 Indoor Outdoor (I/O) Ratios

To explore the protection that buildings provide from outdoor air pollution further we calculated the median I/O ratio. In urban locations the median I/O ratio was 0.92 in the pre‐dry season and 1.0 in the wet season. Median I/O rations were higher in rural locations, being 1.37 in the pre‐dry season and 1.43 in the wet season. I/O ratios >1 may indicate that there are important sources of indoor air pollution in rural areas (e.g., cooking, smoking) during this time. Generally, clean fuels are widely used for cooking, and were reported by the volunteers, but ventilation systems such as extractors were not used by any of the volunteers in our study. In addition, most locations had a smoker present and frequency of smoking was 5–32 cigarettes per day.

During the dry season the median urban I/O ratio was 0.92 and the rural I/O ratio was 1.01. In rural areas, the dry season I/O ratio (1.01) is substantially lower than during the wet season (1.43), indicating that outdoor sources of air pollution became more important. When we only consider fire‐derived PM_2.5_, the median fire‐derived I/O ratio during the dry season was 0.9 across all sites, 0.9 at urban sites and 0.86 at rural sites. Thus, indicating that housing in both urban and rural housing provides little protection from outdoor air pollution. The relatively small reduction in PM_2.5_ from outdoor to indoor environments across all sites is likely due to the typical building design with ventilation above doors and windows, which leads to buildings being poorly sealed from outdoor air pollution.

We compare the mean I/O ratio across Kereng and Palangkaraya in our study (1.01) to previous studies in Indonesia and elsewhere in the world, during wildfire events (Table 2). Ardiyani et al. (2023) reported mean I/O ratios from Palangkaraya of 0.83–1.25, similar to our work. Studies from USA and Canada report I/O ratios of 0.1–1.1. This indicates housing in Indonesia provides less protection from outdoor air pollution than in North America, likely because it is well ventilated and poorly sealed. Houses in this study were typically concrete in urban areas and wooden in rural areas. In both locations houses were well ventilated (poorly sealed), with vents around windows and doors and often open windows. When outdoor air pollution concentrations are lower than indoor concentrations, well‐ventilated houses will help dilute indoor sources of pollution and reduce indoor concentrations. During fire events when outdoor concentrations exceed indoor concentrations well‐ventilated houses allow outdoor pollution sources to enter the home easily. This has implications for the use of HIA in Indonesia, and potentially other locations in the world, where outdoor concentrations are high and housing is poorly sealed. As previously discussed, HIA rely on concentration response functions that are derived from cohort studies, which are heavily biased toward studies from North America and Europe (Burnett et al., 2018; Chen & Hoek, 2020; Pope III et al., 2020) where the I/O ratio is considerably lower than Indonesia. Therefore, the health impacts of air pollution exposure in Indonesia may be underestimated if the concentration response functions were applied to the Indonesian population. Further studies on I/O ratios in Indonesia and other fire‐prone regions in Asia and Africa are needed to determine if the I/O ratios in this study and Ardiyani et al. (2023) are representative of larger regions.

**Table 2:** Comparison of 24‐Hour Indoor Outdoor (I/O) Ratios for This Study and Previous Wildfire Smoke Studies

Study	Location	Year	Time period	Setting	24 hr I/O ratio	
					Mean	Median
This Study	Palangkaraya and Kereng, Indonesia	2023	Fires (Dry Season) (2023‐09‐01 to 2023‐10‐31)	Household	1.01	0.93
Ardiyani et al. (2023)	Palangkaraya, Indonesia	2019	Fires (2019‐08‐01 to 2019‐10‐31)	Household	0.83–1.25	–
He et al. (2022)	Seattle, USA	2020	2020 Washington Wildfire (2020‐09‐07 to 2020‐09‐22)	Household	0.23–0.88	0.21–0.86
Henderson et al. (2005)	Colorado, USA	2002	2002 wildfire season	Household	0.6–1.1	–
Kirk et al. (2018)	Pacific Northwest, USA	2015	Summer 2015	Household	0.1–0.26	
Barn et al. (2008)	British Columbia	2004, 2005	Summer 2004	Household	0.61	
			Summer 2005			
May et al. (2021)	Western USA	2020	September 2020	Household, Commercial, Educational		All: 0.47
						Residential: 0.33
						Commercial: 0.58
						School: 0.78 (All with filter: 0.04)
Stampfer et al. (2024)	Washington, USA	2020–2021	Smoke events between September 2020 and August 2021	Educational		0.22–0.91

### 3.2 Impact of Fires on Daily Mean PM_2.5_ Concentrations

#### 3.2.1 Model Evaluation

Daily‐mean PM_2.5_ concentrations from the outdoor Purple Air sensors were used to evaluate the WRF‐Chem model across Central Kalimantan. We evaluated four model simulations with different FINNpeatSM emissions inputs in order to refine emissions from FINNpeatSM (Table 3).

**Table 3:** Soil Moisture Threshold Combinations Used in FINNpeatSM (High Soil Moisture Threshold, Low Soil Moisture Threshold), and the Corresponding Dry Season (September 1st–October 31st) PM_2.5_ Fire Emissions (Tg), Mean and Maximum Burn Depth (cm) and Daily Mean Model Evaluation Statistics for Each Simulation

Simulation name	Upper soil moisture threshold	Lower soil moisture threshold	Total PM_2.5_ fire emissions (Tg)	Mean/Max burn depth (cm)	Root mean square error (RMSE)	Normalized mean bias fraction (NMBF)	Normalized mean absolute error fraction (NMAEF)
FINNpeatSM_0.5_0.25	0.5	0.25	2.04	23.2/34.0	19.25	0.16	0.18
FINNpeatSM_0.5_0.1	0.5	0.1	1.62	17.9/27.8	14.00	−0.08	0.11
FINNpeatSM_0.45_0.1	0.45	0.1	1.50	16.2/26.6	17.92	−0.15	0.14
FINNpeatSM_0.35_0.1	0.35	0.1	1.20	12.4/23.0	33.05	−0.34	0.22

The location of fires, as detected by fire hotspots, is available at daily resolution from FINN. In FINNpeatSM, where a fire occurs on peatland, peat burn depth is scaled between a minimum and maximum threshold relative to soil moisture. Soil moisture data is provided at 9 km spatial resolution and 6 hourly time resolution, which we average to daily mean. Therefore, peat fire emissions can vary each day at 9 km spatial resolution, dependent upon the soil moisture of peatland (and therefore burn depth) (Equation 2). An upper soil moisture threshold was used to determine the minimum burn depth (5 cm) and a lower soil moisture threshold was used to determine the maximum burn depth (37 cm). Between these soil moisture thresholds, burn depth was assumed to increase linearly with decreasing soil moisture. Therefore, the upper and lower soil moisture threshold will control the burn depth and the total emissions. We created multiple different combinations of upper and lower soil moisture thresholds (Table 3), which result in PM_2.5_ fire emissions with different magnitudes (Table 3, Figure S2 in Supporting Information S1). These emissions were used to model ambient outdoor PM_2.5_ concentrations and are subsequently evaluated by comparing the daily mean modeled PM_2.5_ concentrations to Purple Air observed outdoor daily mean PM_2.5_ concentrations. Daily mean modeled PM_2.5_ concentrations were evaluated using root mean square error (RMSE), normalized mean bias fraction (NMBF) and normalized mean absolute error fraction (NMBF).

Daily PM_2.5_ fire emissions for all soil moisture thresholds in 2023 indicate fire emissions peak in the dry season when the mean soil moisture is lowest (Figure S2 in Supporting Information S1). Fire PM_2.5_ emissions vary from 1.20 to 2.04 Tg dependent upon the upper and lower soil moisture thresholds chosen (Table 3, Figure S2 in Supporting Information S1).

When modeled PM_2.5_ concentrations are compared to observed PM_2.5_ concentrations, all simulations capture the temporal variability of observations well (*r* > 0.9). However, the simulation with 1.62 Tg PM_2.5_ fire emissions captures observed PM_2.5_ concentrations best, with the lowest RMSE (14.00 μg m^−3^), NMBF (−0.08) and NMAE (0.11) (Table 3). Therefore, we use these emissions as our best approximation of the PM_2.5_ fire emissions for 2023.

A comparison between these 2023 FINNpeatSM estimates with other data sets/years is difficult since the methods for calculating fire emissions vary considerably. The closest comparison we can make is to Kiely et al. (2020) who developed the FINNpeatSM emissions data set. However, it should be noted that, although the method for generating FINNpeatSM has not changed from Kiely et al. (2020), we have updated the soil moisture data set used to scale burn depth, from ESA CCI to SMAP. In line with this, we have also altered the soil moisture thresholds between which burn depth is linearly scaled. Kiely et al. (2020) estimated dry season PM_2.5_ fire emissions for El Niño years between 2004 and 2015. The highest emissions occurred in 2015 (9.4 Tg), which was a very strong El Niño year. Emissions in this study, for 2023 (1.62 Tg), are similar to 2012 and 2014 (both 2.2 Tg), considerably lower than 2015. Thus, we can expect the impacts on air quality in 2023 to be substantially lower than in 2015.

More work is needed to further constrain fire emissions in Indonesia. Many parameters used to generate and model fire emissions for the region remain uncertain. These include, burn depth, the diurnal cycle of fires and the emissions from peat fires that smoulder for multiple days. We have assumed a linear relationship between soil moisture and burn depth. The modeled PM_2.5_ concentrations are in good agreement with observed PM_2.5_ concentrations from September through to mid‐October, suggesting this is a reasonable assumption. However, in late October, modeled PM_2.5_ concentrations overestimate observed PM_2.5_ concentrations suggesting our method does not fully capture the temporal variability in emissions. Data from the Geostationary Environment Monitoring Spectrometer satellite is now available for the region. Geostationary Environment Monitoring Spectrometer provides high temporal and spatial resolution data of aerosol optical depth and could provide further information on many of the parameters, which are currently uncertain.

**Figure 5:**
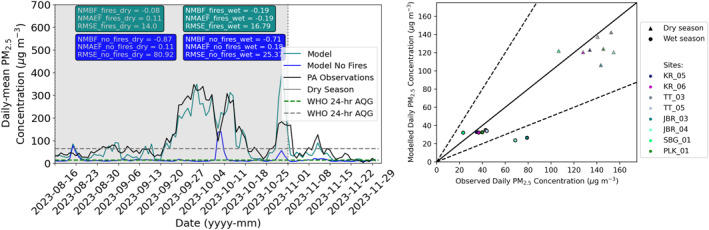
Comparison of observed and modeled PM_2.5_ concentrations (left): Timeseries of modeled daily‐mean PM_2.5_ concentrations with fires (teal) and without fires (blue) compared to observations (black) shown as the average across the 8 outdoor sensors. The World Health Organization 24‐hr guideline limit (15 mg m^−3^) (dashed green line) and the Indonesia 24‐hr guideline limit (65 mg m^−3^) (dashed gray line) and the dry season (gray shading) are indicated. Right: Scatter plot of observed and modeled site mean PM_2.5_ concentrations during the dry season (triangles: August 16th‐1st November 2023) and wet season (circles: November 1st–31st December 2023).

We used WRF‐Chem to simulate ambient outdoor PM_2.5_ concentrations and compare predicted concentrations to outdoor Purple Air sensors (see Table 1 and Figure 5). During the wet season (November 1st to December 31st) the model slightly underpredicts observed concentrations (NMBF: −0.19, 16.8 μg m^−3^) but captures the daily variability well (*r*: 0.79). Fire emissions contribute on average 20 μg m^−3^ (0 μg m^−3^ to 123 μg m^−3^) to simulated PM_2.5_ concentrations during the wet season. Without emissions from fires PM_2.5_ concentrations would lie below the WHO 24‐hr guideline limit at all sites during the wet season.

During the pre‐dry and dry season the model captures the variability in the observed PM_2.5_ concentrations well (*r*: 0.88), and the underestimation of observed concentrations is reduced at most sites (RMSE: 14.0 μg m^−3^, NMBF: −0.08) (Figures 5a and 5b). Modeled PM_2.5_ concentrations without fire emissions are below the WHO 24‐hr guideline limit for most of the pre‐dry and dry season (Figure 5a). This indicates that fires contribute on average 112 μg m^−3^ (93 μg m^−3^ to 131 μg m^−3^) to simulated PM_2.5_ concentrations during the dry season, similar to the contribution estimated using the Purple Air sensors (∼90 μg m^−3^). We also evaluate modeled PM_2.5_ concentrations at a regional scale across Borneo using the government network of reference grade PM_2.5_ sensors (Figure S3 in Supporting Information S1). The model reproduces the variability and magnitude in observed dry season PM_2.5_ concentrations at sites affected by fires well (Figure S3 in Supporting Information S1, further discussion in Supplementary Material). This indicates the model simulates regional increase in ambient PM_2.5_ concentrations across Indonesian Borneo due to the fires well.

### 3.3 Updated Exposure Assessment for Populations in Central Kalimantan for PM_2.5_ From Fires

We analyzed the questionnaire to understand how activities and time spent indoors and outdoors affect the exposure of the population. The questionnaire indicates that all volunteers work in the same village/town that they live in. Therefore, variation in daily exposure to PM_2.5_ is likely to be determined by whether the volunteers are indoors or outdoors rather than their geographical location (i.e., traveling to another village/town for work). Therefore, we focus the exposure adjustment on the amount of time spent indoors and outdoors. To assess indoor and outdoor exposure we grouped volunteers by location and calculated the mean amount of time that volunteers in each location spent indoors and outdoors. The mean amount of time spent indoors and outdoors was relatively homogenous across locations, suggestive that there is little difference in time spent indoors and outdoors between urban and rural locations. In all locations, most of each day is spent indoors (15.4–17.5 hr), and less time spent outdoors (6.5–8.6 hr). We used the mean time spent indoors (16.2 hr) and outdoors (7.8 hr) across all locations to estimate exposure at a regional level.

**Figure 6:**
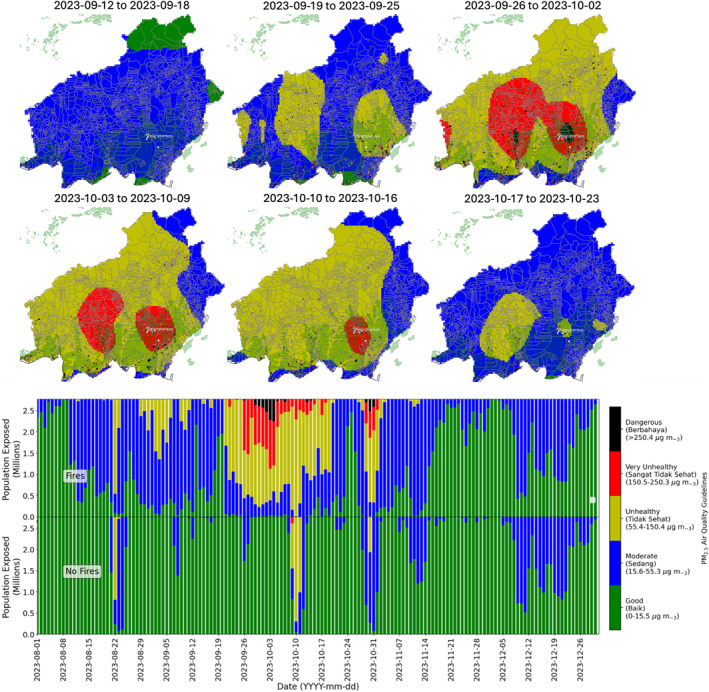
Estimated (top) total daily population exposure to air quality guidelines with and without fires and (bottom) weekly mean exposure to air quality guidelines, both in Central Kalimantan. Additionally shown in panel (b) are the Purple Air sites (white edge color), the location of the capital (Palangkaraya) and MODIS hotspots, for the same week period, colored by fire radiative power (FRP), with darker colors indicating higher FRP.

We estimated exposure to PM_2.5_ from peat‐fires regionally across Central Kalimantan by combining modeled hourly PM_2.5_ concentrations and the mean time spent in indoor and outdoor environments. We use the mean number of hours spent indoors and outdoors across all 3 locations (indoors: 16.2 hr, outdoors: 7.8 hr) and combine this with observed indoor and outdoor hourly‐mean PM_2.5_ concentrations (Equation 5). The widespread degradation in air quality across Central Kalimantan as fires became more frequent through the dry season is clear from Figure 6. During the dry season, PM_2.5_ emissions from fires across Central Kalimantan account for 60% of overall PM_2.5_ concentrations.

Between August 1st to August 10th, when there were few fires, population exposure was dominated by exposure to good and moderate air quality (Figure 6). During this time, 2.65 million people (>95% of the population) were exposed to good (2.1–2.5 million people) or moderate (0–0.6 million people) air quality. In the simulation without fire emissions 100% of the population were exposed to good air quality throughout the same period (Figure 6), indicating that exposure to moderate air quality is due to fire‐derived PM_2.5._ During this time, PM_2.5_ emissions from fires account for 23% of overall PM_2.5_ concentrations.

There was widespread deterioration in air quality from August 11th through to September 18th as fires become widespread and account for 45% of overall PM_2.5_ concentrations. Initially 50%–75% (1.5–2.2 million people) of the total Central Kalimantan population was exposed to moderate air quality (August 11th to August 22nd) (Figure 6). Air quality deteriorated further (August 23rd to September 18th), exposing 10%–70% (0.2–2 million people) of the population to unhealthy air quality on several days. Provinces affected by unhealthy air quality included Kotawaringin Timur, Kotawaringin Barat, Seruyan, Katingan, Kapuas, Pulang Pisau, Barito Selatan and Barito Timur. Exposure to good air quality became much less frequent and only populations in the north‐east and south of the region were exposed to good air quality (including Murung Raya, Barito Utara, southern Katingan, Pulang Pisau and Seruyan).

Air quality deteriorated further from the end of September, as the contribution of PM_2.5_ fire emissions increased to account for 70% of overall PM_2.5_ concentrations, with the highest exposures occurring between September 19th and October 21st (Figure 6). On average 0.07 million people (2.5% of the total population) were exposed to dangerous air quality, and a further 1.55 million people (55% of the total population) were exposed to unhealthy and very unhealthy air quality. Populations in the region capital Palangkaraya, Pulang Pisau, Kapuas, Gunung Mas, Kotawaringin Timur and Seruyan were particularly badly. On the days with the poorest air quality (e.g., October 10th) 0.62 million people (22% of the total population) were exposed to dangerous air quality, and 2.77 million people (>99% of the total population) were exposed to unhealthy and very unhealthy air quality. Without fires there would have been no population exposure to dangerous air quality (Figure 6). Without fire emissions population exposure to unhealthy and very unhealthy air quality would have been substantially reduced, with an average of 0.15 million people (5.3% of the total population) exposed to unhealthy and very unhealthy between September 19th and October 21st. This indicates that fires led to widespread exposure to poor air quality in the region, with ∼1.5 million people (53% of the population) being exposed unhealthy, very unhealthy and dangerous air quality levels due to fires.

Air quality generally improved from October 21st, as the number of fires decreases. However, population exposure to unhealthy, very unhealthy and dangerous air quality continued in Pulang Pisau and Palangkaraya where there were a cluster of fire hotspots. This led to 0.62 million people (23% of the population) being exposed to poor air quality, and on the worst days 2.6 million people exposed (>94% of the population) to unhealthy, very unhealthy or dangerous air quality.

## 4 Conclusions

We deployed a network of 13 low‐cost Purple Air PM_2.5_ sensors across villages in Central Kalimantan, Indonesia, where peat fires are frequent during El Niño conditions. The sensors measured PM_2.5_ concentrations between mid‐August and December 2023, providing measurements of indoor and outdoor PM_2.5_ concentrations through an El Niño dry season (August to October) and the following wet season (November to December). Both indoor and outdoor PM_2.5_ concentrations increased by 90 μg m^−3^ due to smoke from peat fires. Our measurements provide some of the first hourly observations of outdoor and indoor PM_2.5_ concentrations close to Indonesian peat fires.

During the pre‐dry and wet season (non‐fire periods), observed indoor PM_2.5_ concentrations were 40% higher than outdoor PM_2.5_ concentrations in rural locations, leading to I/O ratios >1. This indicates that there are important indoor sources of air pollution (e.g., cooking, smoking) during this time. During the dry season when there are frequent fires, the I/O ratio decreased in both urban and rural locations. We estimated the I/O ratio for fire‐derived PM_2.5_ concentrations across all sites was 0.86–0.9, which indicates that buildings provide little protection from outdoor fire‐derived PM_2.5_. Our results suggest people are exposed to poor air quality both indoors and outdoors and it is difficult for the population to reduce their exposure to PM_2.5_ from fires.

We used a regional air quality model alongside our measurements of outdoor PM_2.5_ concentrations to refine estimates of PM_2.5_ fire emissions from peat fires in FINNpeatSM. In FINNpeatSM, where a fire occurs on peatland, peat burn depth is scaled between a minimum and maximum threshold relative to soil moisture. The relationship between soil moisture and burn depth remains a large uncertainty in fire emissions data set for peat fires. Therefore, we tested various soil moisture thresholds, which altered the minimum and maximum burn depth and the gradient of burn depth. We found that all model simulations capture daily variability in PM_2.5_ concentrations well (*r* = >0.9). But there were differences in how well simulations captured the magnitude of PM_2.5_ concentrations best (RMSE, NMBF, NMAE). Our best estimate (minimum RMSE, NMBF and NMAE) of Indonesian PM_2.5_ fire emissions for the 2023 dry season was 1.62 Tg, indicating fire emissions in 2023 were comparable to 2012 and 2014.

Our updated estimate of population exposure to poor air quality due to fires across Central Kalimantan indicated that during the worst period of air quality (September 19th and October 21st) 0.07 million people (2.5% of the total population) were exposed to dangerous air quality, and a further 1.55 million people (55% of the total population) were exposed to unhealthy and very unhealthy air quality. This indicates that exposure to poor air quality during fire periods is widespread across the region, and the health impacts are likely to be substantial. Our estimates are evaluated against measurements of PM_2.5_ concentrations from both low‐cost sensors and the government network of reference grade sensors, adding confidence to our results.

The implications of our findings underscore the need for cross‐sectoral policy and governance reform targeting the root cause of peatland fires (Evers et al., 2017; Padfield et al., 2014), as well as targeted public health policies that adapt to dynamic seasonal air pollution. Housing designs in Kalimantan tend to be well ventilated to the outdoors and provide little protection from outdoor air pollution. Building designs that improve the sealing of homes in urban areas could reduce the penetration of fire‐derived PM_2.5_. Simultaneously, our findings highlight the need for efforts to reduce indoor pollution sources in rural areas (e.g., due to cooking), which consistently elevate indoor exposure. Clean fuels are widely used for cooking but ventilation systems such as extractors were not used by any of the volunteers in our study. These could help to alleviate indoor exposure during cooking. Although well‐ventilated houses help reduce exposure to indoor pollution sources they provide little protection when outdoor air pollution during fire haze episodes. Our findings also challenge existing health impact assessment models that are often based on studies from regions with lower I/O ratios, suggesting that localized assessments are essential for accurately estimating the health impacts of PM_2.5_ exposure in Indonesia and other regions affected by smoke from fires.

## Conflict of Interest

The authors declare no conflicts of interest relevant to this study.

## Supporting information

Supporting Information S1

## Data Availability

Government observations of PM_2.5_ across Indonesia were accessed from the https://www.bmkg.go.id/kualitas‐udara/informasi‐partikulat‐pm25.bmkg. The authors acknowledge use of the Weather Research and Forecasting model coupled with Chemistry preprocessor tool mozbc, fire_emiss, bio_emiss and anthro_emis provided by the Atmospheric Chemistry Observations and Modeling Lab (ACOM) of NCAR. As far as we are aware there are no competing financial interests. Code to setup and run WRFChem (using WRFotron version 2.0) are available through Conibear and Knote (2020). Model simulation data and PM_2.5_ observations from the Purple Airs are available at Graham and Spracklen (2024) and Graham et al. (2024).
