# Supplementary material for: Updated Smoke Exposure Estimate for Indonesian Peatland Fires Using a Network of Low‐Cost PM2.5 Sensors and a Regional Air Quality Model
Source: Geohealth. 2024 Nov 3;8(11):e2024GH001125. doi: 10.1029/2024GH001125 (PMC11532237; doi:10.1029/2024GH001125)
Supplement: Supplementary file 1 — Supporting Information S1 [file GH2-8-e2024GH001125-s001.pdf]

GeoHealth

Supporting Information for

Supplementary Material: Updated Smoke Exposure Estimate for Indonesian Peatland Fires using a Network of Low-cost Purple Air $PM_{2.5}$ sensors

Ailish M Graham*[1,2], Dominick V Spracklen[1], James B McQuaid[1], Thomas E L Smith[3], Hanun Nurrahmawati[4], Devina Ayona[4], Hasyim Mulawarman[5], Chaidir Adam[5], Effie Papargyropoulou[1], Richard Rigby[1], Rory Padfield[1], Shofwan Choiruzzad[4]

1. School of Earth and Environment, University of Leeds, UK
2. National Centre for Earth Observation, University of Leeds, UK
3. Department of Geography and Environment, London School of Economics and Political Science, UK
4. Department of International Relations, Universitas Indonesia, Indonesia
5. University of Palangkaraya, Indonesia

Contents of this file

Text

Text S1: Purple Air Evaluation
Text S2: Fire Emission Evaluation
Text S4: Spatial Fire Emissions

Figures

Figure S1: Comparison of Purple Air with Reference Grade measurements of daily-mean $PM_{2.5}$ concentrations. Timeseries of Purple Air daily-mean $PM_{2.5}$ concentrations (green) compared to Reference Grade measurements of daily-mean $PM_{2.5}$ concentrations (black). The World Health Organisation 24-hour guideline limit (15 mg m^{-3}) (dashed green line) and the Indonesia 24-hour guideline limit (65 mg m^{-3}) (dashed grey line) and the dry season (grey shading) are indicated.

Figure S2: (a) Daily $PM_{2.5}$ fire emissions with varying soil moisture threshold combinations. The upper and lower soil moisture thresholds chosen determine the burn depth of peat fires. The upper soil moisture threshold determines the soil moisture below which burn depth is >5 cm. While the lower soil moisture threshold determines the soil moisture at which burn depth reaches its maximum (37 cm). FINNpeatSM_0.5_0.25: FINNpeatSM with upper SM threshold: 0.5 m^3 m^{-3} and lower SM threshold: 0.25 m^3 m^{-3}, FINNpeatSM_0.5_0.1: FINNpeatSM with upper SM threshold: 0.5 m^3 m^{-3} and lower SM threshold: 0.1 m^3 m^{-3}, FINNpeatSM_0.45_0.1: FINNpeatSM with upper SM threshold: 0.45 m^3 m^{-3} and lower SM threshold: 0.1 m^3 m^{-3} and FINNpeatSM_0.35_0.1: FINNpeatSM with upper SM threshold: 0.35 m^3 m^{-3} and lower SM threshold: 0.1 m^3 m^{-3}. FINN_nrt with no peat burning (gray dashed) is also shown for reference. SMAP daily mean soil moisture (dotted blue) across Indonesian peatland during 2023 is also shown. The dry season is indicated by grey shading.

Figure S3: Comparison of observed and modelled $PM_{2.5}$ concentrations across Indonesian Borneo. Timeseries of modelled daily-mean $PM_{2.5}$ concentrations with fires (teal) and without fires (blue) compared to observations (black), at each of the 7 governmental observation sites in Indonesian Borneo. The World Health Organisation 24-hour guideline limit (15 mg m-3) is indicated (dashed green line) and the Indonesia 24-hour guideline limit (65 mg m-3) (dashed grey line) and the dry season is indicated in (grey shading) are indicated. Inset map: modelled (contour) and observed (scatter) fire-derived dry season (September 1st to October 31st) mean $PM_{2.5}$ concentrations, calculated as the dry season mean minus the wet season (November 1st to December 1st) mean.

Figure S4: FINNpeatSM_0.5_0.1 dry season (September 1st to October 31st 2023) $PM_{2.5}$ fire emissions (Gg) across the field study domain (1 km resolution) and Indonesian Borneo (10 km resolution). Central Kalimantan is indicated by grey shading and peatland is shaded in green. The locations of the Purple Air sensors is also indicated by coloured circles.

Tables

Table S1: Participant questionnaire included in this study which each participant completed.

Introduction

Additional information includes:

- The evaluation of Purple Air sensors using a reference grade governmental site in Palangkaraya.
- Detailed analysis of $PM_{2.5}$ emissions from fires.
- Detailed comparison of modelled $PM_{2.5}$ concentrations across Borneon Indonesia using network of government reference grade sensors.
- Spatial maps of fire emissions used in this study.
- Blank questionnaire used to collect information on time spend indoors/outdoors for exposure estimation.

This information supports the analysis presented in the manuscript but is not essential to a reader. Therefore, we provide it separately for those who may want to know more about our method or the analysis that supports the results presented.

Purple Air Evaluation

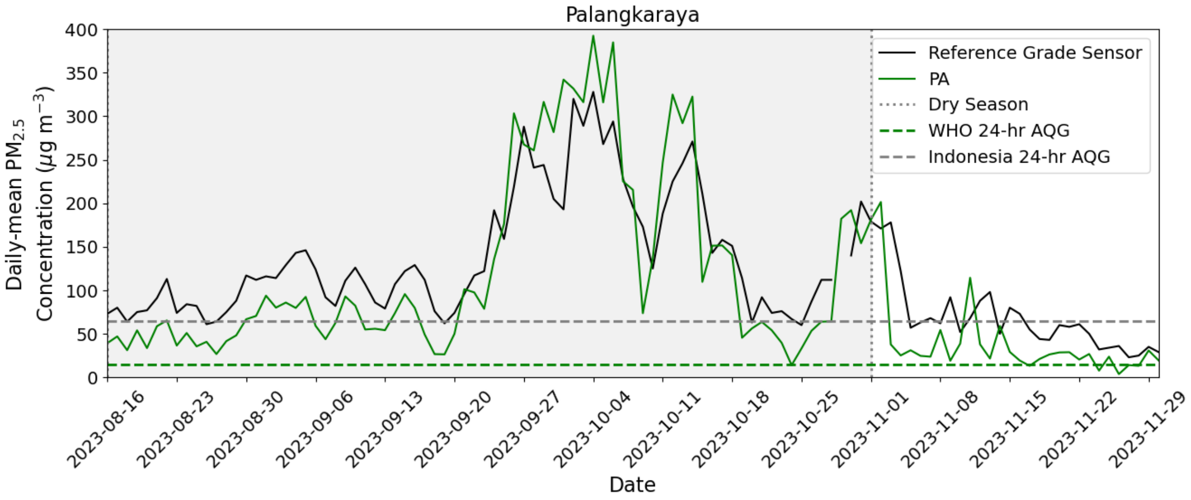

Figure S1: Comparison of Purple Air with Reference Grade measurements of daily-mean $PM_{2.5}$ concentrations. Timeseries of Purple Air daily-mean $PM_{2.5}$ concentrations (green) compared to Reference Grade measurements of daily-mean $PM_{2.5}$ concentrations (black). The World Health Organisation 24-hour guideline limit (15 μg m^{-3}) (dashed green line) and the Indonesia 24-hour guideline limit (65 μg m^{-3}) (dashed grey line) and the dry season (grey shading) are indicated.

Text S1: We compared daily-mean $PM_{2.5}$ concentrations from the outdoor Purple Air sensor located in Palangkaraya (PA01) to a reference grade sensor in Palangkaraya located within 2 km. The PA and reference sensor compare well (r=0.92, NMBF=0.01) (Figure S1) particularly during the dry season (NMBF: 0.05) but the PA sensor measures lower $PM_{2.5}$ concentrations, in the wet season (NMBF: -0.16). The higher $PM_{2.5}$ concentrations measured by the reference grade sensor may partly be due to its urban location, compared to the suburban location of the PA. During the dry season, when $PM_{2.5}$ is dominated by smoke from fires, $PM_{2.5}$ concentrations are controlled by regional transport of smoke so there is less local variability in $PM_{2.5.}$ Overall, we find the Purple Air adequately captures $PM_{2.5}$ concentrations, when compared with a reference grade sensor.

Model Evaluation

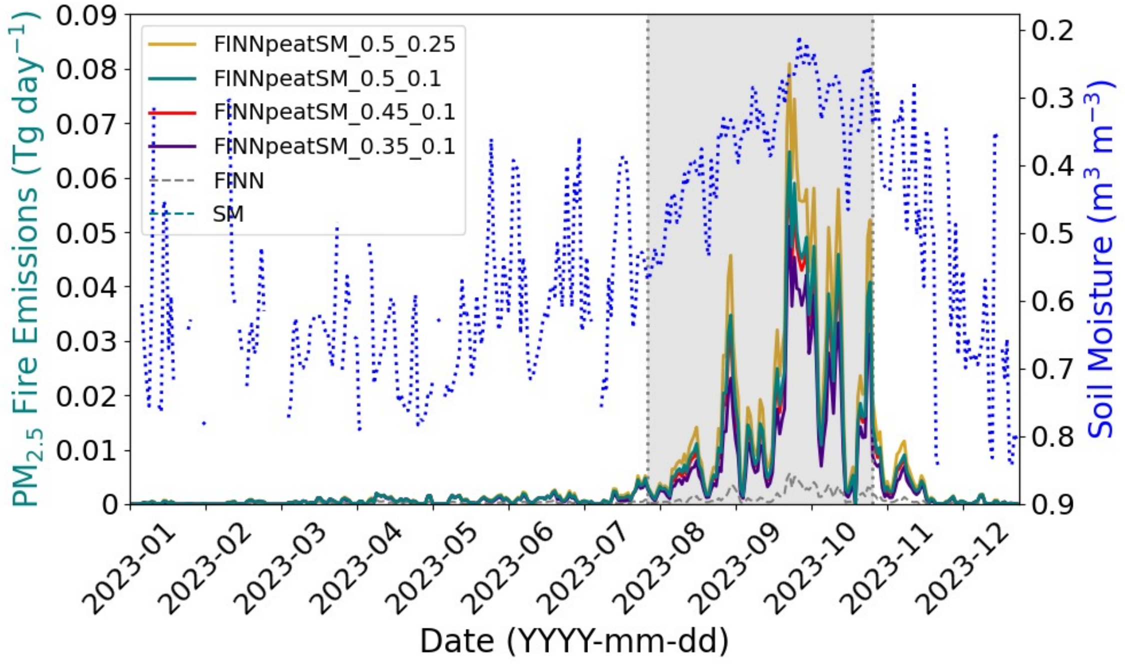

Figure S2. (a) Daily $PM_{2.5}$ fire emissions with varying soil moisture threshold combinations. The upper and lower soil moisture thresholds chosen determine the burn depth of peat fires. The upper soil moisture threshold determines the soil moisture below which burn depth is >5 cm. While the lower soil moisture threshold determines the soil moisture at which burn depth reaches its maximum (37 cm). FINNpeatSM_0.5_0.25: FINNpeatSM with upper SM threshold: 0.5 m^3 m^{-3} and lower SM threshold: 0.25 m^3 m^{-3}, FINNpeatSM_0.5_0.1: FINNpeatSM with upper SM threshold: 0.5 m^3 m^{-3} and lower SM threshold: 0.1 m^3 m^{-3}, FINNpeatSM_0.45_0.1: FINNpeatSM with upper SM threshold: 0.45 m^3 m^{-3} and lower SM threshold: 0.1 m^3 m^{-3} and FINNpeatSM_0.35_0.1: FINNpeatSM with upper SM threshold: 0.35 m^3 m^{-3} and lower SM threshold: 0.1 m^3 m^{-3}. FINN_nrt with no peat burning (gray dashed) is also shown for reference. SMAP daily mean soil moisture (dotted blue) across Indonesian peatland during 2023 is also shown. The dry season is indicated by grey shading.

Text S2: Daily FINNpeatSM $PM_{2.5}$ fire emissions for all soil moisture thresholds in 2023 indicate fire emissions peak in the dry season when the mean soil moisture is lowest (Figure S2). During the dry season the mean soil moisture is 0.48 m^3 m^{-3} (mean anomaly of -0.1 m^3 m^{-3}). The mean and maximum burn depth, and therefore total fire $PM_{2.5}$ emissions, vary substantially dependent upon the upper and lower soil moisture thresholds chosen. When modelled $PM_{2.5}$ concentrations are compared to observed $PM_{2.5}$ concentrations all simulations capture the temporal variability of observations well ($r > 0.9$). However, FINNpeatSM_0.5_0.25 overestimates observed $PM_{2.5}$ concentrations (RMSE = 19.25, NMBF = 0.16, NMAEF = 0.18), particularly later in the dry season. In contrast, FINNpeatSM_0.5_0.1, FINNpeatSM_0.45_0.1 and FINNpeatSM_0.35_0.1 generally underestimate observed $PM_{2.5}$ concentrations between September 27th and October 25th, but the overestimation between October 25th and October 31st seen in FINNpeatSM_0.5_0.25 is reduced. $PM_{2.5}$ emissions are lowest in FINNpeatSM_0.35_0.1 (1.20 Tg), due to low mean and maximum burn depths (12.4 cm and 23 cm), which underestimates $PM_{2.5}$ concentrations most (NMBF = -0.34). For FINNpeatSM_0.5_0.1 and FINNpeatSM_0.45_0.1, total $PM_{2.5}$ emissions (1.62 Tg and 1.50 Tg, respectively) and mean/maximum burn depth (17.9/27.8 cm and 16.2/26.6 cm, respectively) are very similar. However, FINNpeatSM_0.5_0.1 captures observed $PM_{2.5}$ concentrations best of all the simulations. FINNpeatSM_0.5_0.1 has the lowest RMSE (14.00 mg m^{-3}), NMBF (-0.08) and NMAE (0.11) (Table 3). Therefore, we use the FINNpeatSM_0.5_0.1 as our best approximation of the $PM_{2.5}$ fire emissions for 2023.

FINNpeatSM_0.5_0.1 dry season fire emissions are 1.62 Tg. Daily fire emissions peak at 0.06 Tg day^{-1} on 28th September, when mean soil moisture was very low (0.44 m^3 m^{-3}) (anomaly of -0.13 m^3 m^{-3}). Daily fire emissions remain high into October when monthly fire emissions are highest (0.84 Tg $month^{-1}$) and soil moisture remains low. Minimum soil moisture was 0.43 m^3 m^{-3} on October 4th (anomaly of -0.14 m^3 m^{-3}). In contrast, FINN emissions (without peat) peak at 0.006 Tg day^{-1} on 28th September, indicating high emissions in FINNpeatSM are dominated by peat combustion due to low soil moisture. Total $PM_{2.5}$ emissions in FINN between August and October are 0.14 Tg and total emissions in October are 0.07 Tg $month^{-1}$, >10 ´ lower than FINNpeatSM.

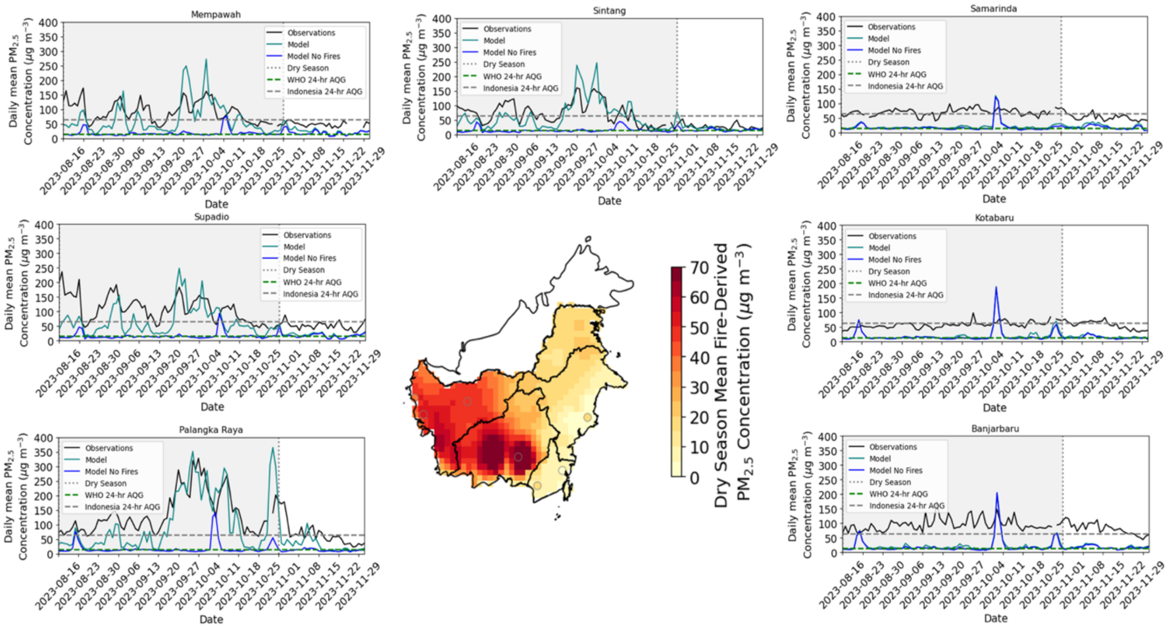

Figure S3. Comparison of observed and modelled $PM_{2.5}$ concentrations across Indonesian Borneo. Timeseries of modelled daily-mean $PM_{2.5}$ concentrations with fires (teal) and without fires (blue) compared to observations (black), at each of the 7 governmental observation sites in Indonesian Borneo. The World Health Organisation 24-hour guideline limit (15 μg m-3) is indicated (dashed green line) and the Indonesia 24-hour guideline limit (65 μg m-3) (dashed grey line) and the dry season is indicated in (grey shading) are indicated. Inset map: modelled (contour) and observed (scatter) fire-derived dry season (September 1st to October 31st) mean $PM_{2.5}$ concentrations, calculated as the dry season mean minus the wet season (November 1st to December 1st) mean.

115

2023 Dry Season Fire Emissions

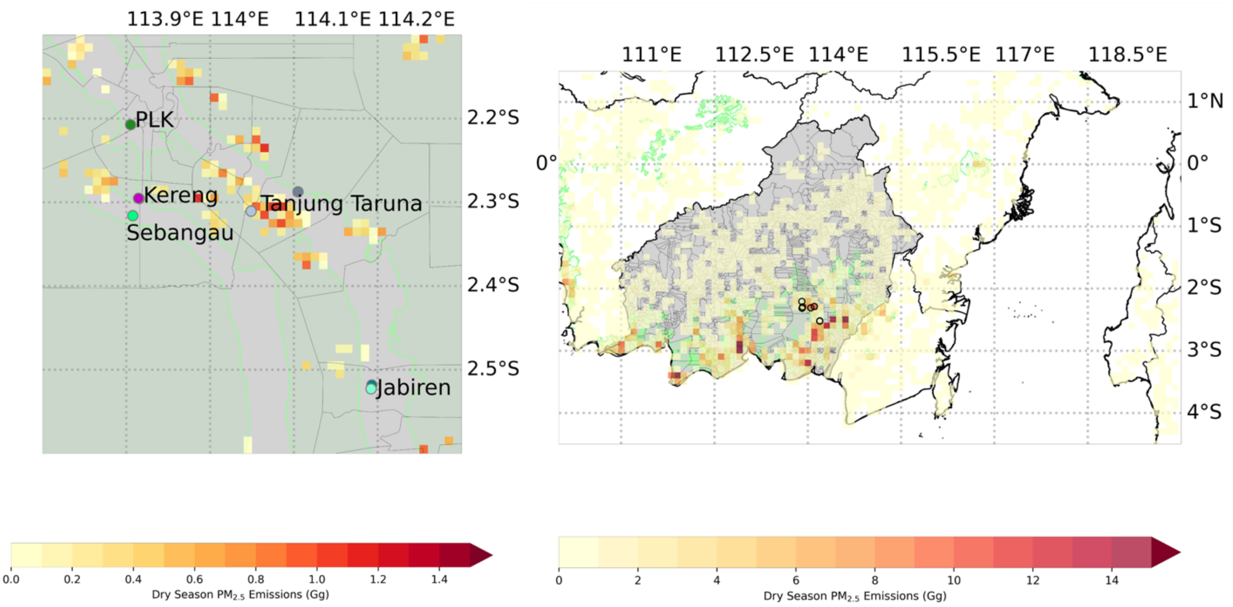

Figure S4. FINNpeatSM_0.5_0.1 dry season (September 1st to October 31st 2023) $PM_{2.5}$ fire emissions (Gg) across the field study domain (1 km resolution) and Indonesian Borneo (10 km resolution). Central Kalimantan is indicated by grey shading and peatland is shaded in green. The locations of the Purple Air sensors is also indicated by coloured circles.

Text S4: Figure S4 indicates the total dry season $PM_{2.5}$ emissions (Gg) across the field study domain and Indonesian Borneo. The highest $PM_{2.5}$ fire emissions are observed in Pulang Pisau to the south of Palangkaraya in Central Kalimantan. Fire emissions in Pulang Pisau are particularly high in the are surrounding Tanjung Taruna and Jabiren. Pulang Pisau is dominated by deep peatlands (green shading), that have been drained for agriculture, leading to low soil moisture and therefore fires that burn deep into the peat below, leading to high fire $PM_{2.5}$ emissions.

130

Participant Questionnaire

Table S1. Participant questionnaire included in this study which each participant completed.

Date	Surveyor	Village

Demography			
Code	**Instructions**	***Format***	**Answer**
idsubid	Personal unique identification number	*Code ID number*	
idname	What is your first name?	*Text*	
idphone	What is your cellphone/WhatsApp number?	*Number*	
idinternetaccess	Does your handphone have access to the Internet?	*0: No*<br>*1: Yes*	
idwifi	Do you have Wi-Fi/Orbit at home?	*0: No*<br>*1: Yes*	
idelectricitynumber	Tell us your electricity token customer number!	*Number*	
idrole	Position of the person answering the survey?	*Text. Note down the position of the person especially representative of organization. Including village head, teachers in the village. Tlk_Position (to code)*	
idsex	What is the sex of the person?	*1: Male*<br>*2: Female*	
ideducation	Have you ever attended school?	*0: No*<br>*1: Yes*	
idedu	What is the max school grade that you have completed?	*0: no education*<br>*1-9: SD,SMP Grade*<br>*10-12: SMA*<br>*13: anything beyond high school*	
idhousetype	What material are your house outer walls built of?	*1: Wood*<br>*2: Bamboo*<br>*3: Brick*<br>*4: Cement*	
idflooring	What material is your house floor built of?	*1: Wood*<br>*2: Tiles*<br>*3: Metal*<br>*4: Concrete/Asbestos*<br>*5: Stone*<br>*5: Other*	
idhousesize	How many bedrooms are there in your house?	*1: 1*<br>*2: 2*<br>*3: 3*<br>*4: 4*<br>*5: 5+*	
idemployed	Do you have an income?	*0: No*<br>*1: Yes*	

idlivelihood	What are your main livelihood activities? e.g., subsistence farming, sell agriculture commodity, labor worker, government official, (allow them to answer openly, listen kindly, and based on their answer, list up to three)	*Text*	
idliv1	Livelihood 1	*Tlk_Livelihood (to code)*	
	Are there other sources of income?		
idliv2	Livelihood 2	*Tlk_Livelihood*	
idliv3	Livelihood 3	*Tlk_Livelihood*	
idhoursliv	In the past 48 hours, how many hours have you spent at each livelihood?	*Text (idliv1: hours)*<br>*Text (idliv2: hours)*<br>*Text (idliv3: hours)*	
idagr	Do you practice agriculture?	*0: No*<br>*1: Yes*	
idtimehomework	You have 24 pebbles to represent a day (1 pebble per hour). Could you show us on average, how many hours you spend at home and how many at each livelihood?	*Home: Pebble number*<br>*Livelihood1: Pebble number*<br>*Livelihood2: Pebble number*<br>*Livelihood3: Pebble number*	
idhoursindooroutdoorhome	You had X pebbles at home. Now can you show me how many pebbles you spent inside and outside when at home?	*Inside: Pebble number*<br>*Outside: Pebble number*	
idhoursindooroutdoorlivelihood 1	You had X pebbles at livelihood1. Now can you show me how many of those X pebbles you spent inside and outside when at livelihood1?	*Inside: Pebble number*<br>*Outside: Pebble number*	
Idhoursindooroutdoorlivelihood 2	You had X pebbles at livelihood2. Now can you show me how many of those X pebbles you spent inside and outside when at livelihood2?	*Inside: Pebble number*<br>*Outside: Pebble number*	
Idhoursindooroutdoorlivelihood 3	You had X pebbles at livelihood3. Now can you show me how many of those X pebbles you spent inside and outside when at livelihood3?	*Inside: Pebble number*<br>*Outside: Pebble number*	
idexpense	How much is your house bill in 1 month?	*1: Less than 1 million*<br>*2: 1-2 million*<br>*3: 2-3 million*<br>*4: 3-4 million*<br>*5: more than 4 million*	
**Indoor Exposure**			
idaircirc	Does your house have air conditioning, fan or neither?	*1: aircon*<br>*2: fan*<br>*0: neither*	
idwindows	Do the windows on the outside of your house open and close?	*0: No*<br>*1: Yes*	
iddoors	Do the doors on the outside of your house open and close?	*0: No*<br>*1: Yes*	
idsmoker	Do you smoke?	*0: No 1: Yes*	
idsmokefreq	If yes, how many times per day?	*Answer: Number*	
idpassivesmoker	Does anyone in your household smoke?	*0: No 1: Yes*	

idpassivesmokefreq	If yes, how many times per day?	*Answer: Number*	
idcook	Do you cook?	*0: No*<br>*1: Yes*	
idcookfreq	If yes, how many times per day on average?	*Answer: Number*	
idcookingfuel	What fuel do you use to cook with?<br><br>*n.b. Participants classed as 'exclusive clean cooking fuel' if they report using clean fuel regularly AND reported no use/rare use (holidays/guests staying) of solid fuel.*	*1: Coal, wood, agriculture residue, peat (solid fuel stoves)*<br>*2: Gas*<br>*(clean fuel stoves)*<br>*3: Electricity*<br>*(clean fuel stoves)*<br>*4: Other (text details)*	
idcookinglocation	Do you cook indoors or outdoors?	*1. Indoors*<br>*2. Outdoors*	
idcookingventillation	If you cook indoors, do you use any of the following ventilation systems? You can pick multiple answers	*1: Extractor Fan*<br>*2: Open exterior window*<br>*3: Open exterior door*<br>*4: None*	
idmosquitocoils	Do you use mosquito coils in your house?	*0: No*<br>*1: Yes*	
idcandles	Do you use candles in your house?	*0: No*<br>*1: Yes*	
idincence	Do you use incense in your house?	*0: No*<br>*1: Yes*	
**Outdoor Exposure**			
idworkplace	Is your workplace in this village?	*0: No*<br>*1: Yes*	
idworkplacename	If no, which village is it in?	*Text (village name)*	
idhourstrav	How long does it take you to travel to your workplace in minutes?	*Text (minutes)*	
idtrav	How do you travel to your workplace?	*1: Car*<br>*2: Motorcycle*<br>*3: Boat*<br>*4: Bicycle*<br>*5: Walk*<br>*6: Other (text details)*	
**Exposure and exposure reduction strategies**			
idfirefight	Do you participate as fire team e.g., (Regu Pemadam Kebakaran = RPK) or community groups for fire management (Kelompok Masyarakat Pengendali Kebakaran = KMPK?	*0: No*<br>*1: Yes*	
id_haze	Since living here, have you been impacted by air pollution from peat fires?	*0: No*<br>*1: Yes*	
id_haze_impact	Can you explain how haze from peat fires has impacted you?		
Id_coping_strategy	How have you managed with the impact e.g., coping, adjusting, other strategy? Explain…	*(to code)*	
